# Identification of a c-MYB-directed therapeutic for acute myeloid leukemia

**DOI:** 10.1038/s41375-022-01554-9

**Published:** 2022-04-02

**Authors:** Katherine Clesham, Vanessa Walf-Vorderwülbecke, Luca Gasparoli, Clemence Virely, Sandra Cantilena, Alexia Tsakaneli, Sarah Inglott, Stuart Adams, Sujith Samarasinghe, Jack Bartram, Gareth Williams, Jasper de Boer, Owen Williams

**Affiliations:** 1 Cancer Section, Developmental Biology and Cancer Programme, UCL Great Ormond Street Institute of Child Health, London, UK; 2 SIHMDS-Haematology, Great Ormond Street Hospital for Children, London, UK; 3 Department of Paediatric Haematology, Great Ormond Street Hospital for Children, London, UK; 4 Wolfson Centre for Age-Related Diseases, King’s College London, London, UK; 5 Present Address: University College London Hospital, London, UK

**Keywords:** Preclinical research, Targeted therapies

## Abstract

A significant proportion of patients suffering from acute myeloid leukemia (AML) cannot be cured by conventional chemotherapy, relapsed disease being a common problem. Molecular targeting of essential oncogenic mediators is an attractive approach to improving outcomes for this disease. The hematopoietic transcription factor c-MYB has been revealed as a central component of complexes maintaining aberrant gene expression programs in AML. We have previously screened the Connectivity Map database to identify mebendazole as an anti-AML therapeutic targeting c-MYB. In the present study we demonstrate that another hit from this screen, the steroidal lactone withaferin A (WFA), induces rapid ablation of c-MYB protein and consequent inhibition of c-MYB target gene expression, loss of leukemia cell viability, reduced colony formation and impaired disease progression. Although WFA has been reported to have pleiotropic anti-cancer effects, we demonstrate that its anti-AML activity depends on c-MYB modulation and can be partially reversed by a stabilized c-MYB mutant. c-MYB ablation results from disrupted HSP/HSC70 chaperone protein homeostasis in leukemia cells following induction of proteotoxicity and the unfolded protein response by WFA. The widespread use of WFA in traditional medicines throughout the world indicates that it represents a promising candidate for repurposing into AML therapy.

## Introduction

Acute myeloid leukemia (AML) is an aggressive disease with poor outcomes in both young and older patients, despite the improved application of intensive chemotherapy, risk stratification and hematopoietic stem cell transplantation [1, 2]. Unacceptable toxicities limit the scope for further escalation of chemotherapy, with further improvements in outcome likely to come from more selective therapies such as molecular targeting of disease-specific pathways and oncogenic drivers. The latter has had spectacular success in specific subtypes of leukemia, exemplified by arsenic trioxide/all-trans retinoic acid induced degradation of the PML/RARA fusion protein in acute promyelocytic leukemia and imatinib inhibition of BCR/ABL fusion protein kinase activity in chronic myeloid leukemia [3, 4].

The potential benefits of harnessing this approach to identify novel leukemia drug-susceptibilities has led to extensive research into the mechanisms maintaining disease in different molecular subtypes of AML. Of particular note is the detailed characterization of MLL-fusion protein-induced dysregulation of epigenetic machinery controlling transcription of critical target genes in *MLL*-rearranged leukemia [5–8]. This has led to the identification of several critical components of aberrant MLL-fusion protein complexes that are amenable to pharmacological targeting [9, 10]. Further research has shown that the transcription factor c-MYB [11] is responsible for mediating the leukemogenic activity of MLL-fusion proteins in AML [12–15]. A recent study suggests that c-MYB plays a central downstream role in integrating the activities of oncogenic drivers in a broad range of different AML subtypes, as well as *MLL*-rearranged AML [16]. This suggests that c-MYB represents a therapeutic target of great relevance to AML in general.

An important potential obstacle to targeting c-MYB in AML is its essential function in normal hematopoiesis [17–19]. This may be mitigated by the increased dependency of leukemia cells to c-MYB expression [20, 21] and by inhibition of mechanisms regulating the expression and function of c-MYB critical in AML but to a lesser extent in normal hematopoietic cells [22]. An example of the latter is pharmacological inhibition of the interaction between c-MYB and the CBP/P300 transcriptional co-activator complex, essential for induction and maintenance of AML [16, 23–25].

We previously reported screening the Connectivity Map (CMAP) database with an AML-derived c-MYB gene expression signature to identify the anti-helminth drug mebendazole as a candidate for repurposing into AML therapy, by virtue of inducing proteasomal degradation of the c-MYB protein [26]. Here, we report that another drug identified by the CMAP screen also targets c-MYB and exhibits potent anti-AML activity. Withaferin A (WFA) is a steroidal lactone found in medicinal extracts of the Indian ginseng plant Withania somnifera and is widely used in traditional medicine as well as a dietary supplement [27]. Exposure of AML cells to WFA caused rapid loss of c-MYB protein, accompanied by activation of the unfolded protein response (UPR) and heat shock response pathways, resulting in inhibition of AML cell growth and self-renewal, and impaired AML progression in vivo. In contrast, WFA spared colony formation by normal hematopoietic progenitor cells. This work highlights a novel approach to targeting c-MYB in AML and identifies WFA as a potential anti-AML therapeutic.

## Materials and methods

### Mice

Mice were maintained in the UCL GOSICH animal facilities and experiments were performed according to and approved by the United Kingdom Home Office regulations and followed UCL GOSICH institutional guidelines.

### AML PDX samples

Ethical approval was given (Research Ethics Committee reference 14/EM/0134) for use of appropriately consented material from patients with AML at Great Ormond Street Hospital for Children (London, UK). 1-2 × 10^6^ mononuclear cells (Supplementary Table 1) were intravenously or intra-bone injected into 2 G irradiated 5- to 12-week-old NSG mice. Recipient mice were sacrificed upon developing clinical signs of disease. Human AML PDX cells were harvested and purified from bone marrow using the mouse cell depletion kit (Miltenyi Biotec, Surrey, UK).

### Cell culture and reagents

Human AML cell lines were purchased from the ATCC (THP1) and DSMZ (OCI-AML3, U937, MV4;11 and SHI-1), authenticated by short tandem repeat profiling using the PowerPlex 16 system (Promega, Southampton, UK) and mycoplasma negative status confirmed using the MycoAlert Mycoplasma Detection Kit (Lonza, Verviers, Belgium). The following reagents and inhibitors were used, Withaferin A (Merck Life Science UK, Dorset, UK and Cayman Chemical, Ann Arbor, MI, United States); Cyclohexamide, Thapsigargin and MG132 (Merck Life Science UK); Actinomycin D (Cambridge Bioscience, Cambridge, UK); pifithrin-μ. (Abcam, Cambridge, UK).

### Colony formation assays

AML cell lines were plated in HSC002 (Bio Techne, Abingdon, UK), normal CD34^+^ cord blood-derived cells (ZenBio, NC, USA) in HSC005 (Bio Techne) and primary AML cells in HSC005 methylcellulose medium supplemented with 50 ng/ml TPO and FLT3L (PeproTech, London, UK). Colony number was determined and morphology scored 14 days later.

### RNA sequencing (RNA-seq) and Gene set enrichment analysis (GSEA)

Total cellular RNA was purified from control and WFA treated samples from three independent experiments and submitted to UCL Genomics for RNA-sequencing, as detailed in Supplementary Materials and Methods. GSEA (https://software.broadinstitute.org/gsea/) was used to examine enrichment of gene sets for c-MYB target genes (bound by c-MYB in mouse myeloid ERMYB cells [28] and deregulated in THP1 cells following siRNA mediated c-MYB silencing [29]), gene expression changes following shRNA [15], CRISPR-mediated [30] and peptidomimetic [25] c-MYB targeting, AML LSC [13], PMA-induced myeloid differentiation [29, 31], C/EBPβ target genes [31] and the c2.cp.reactome.v7.4.symbols.gmt and c5.go.bp.v7.4.symbols.gmt collection of gene sets from the MSigDB database (http://www.gsea-msigdb.org/gsea/msigdb/index.jsp), in gene expression changes resulting from 6 h exposure of THP1 cells to 1 μM WFA or DMSO (Geo repository: GSE178940). Interrogation of the CMAP database (https://portals.broadinstitute.org/cmap/) [32] using the SPIEDw web tool (http://www.spied.org.uk/) [33] with an AML c-MYB gene expression signature was previously reported [26].

### In vivo transplantation

Luciferase expressing THP1 cells were transplanted into non-irradiated NOD-SCID-γ^-/-^ (NSG; The Jackson Laboratory, Bar Harbor, ME, USA) mice. Recipient mice were imaged using the IVIS® Lumina Series III (PerkinElmer, Beaconsfield, UK) and randomly allocated to control or WFA-treated groups, by flipping a coin. WFA (40 mg/kg of diet) was administered *ad libitum* in regular powdered diet, changed daily. No blinding was used.

### Lentivirus vector constructs

Generation of the pCSGW-PIG and pCSGW-PIG-ΔMYB lentivirus expression constructs was described previously [26].

### Western blot and co-immunoprecipitation (Co-IP) analysis

Western blot and Co-IP analyses were performed as previously described [26] and as detailed in Supplementary Materials and Methods.

### Quantitative RT-PCR (qRT-PCR) analysis

Quantitative RT-PCR (qRT-PCR) was performed on isolated mRNA using TaqMan probe-based chemistry, as previously described [26], using a StepOnePlus Real-Time PCR System (Thermo Fisher Scientific). All primer/probe sets were from Applied Biosystems, Life Technologies.

### Statistics

Statistical significance was determined using Prism (GraphPad) software. Statistical analysis of means was performed using the one sample *t*-test or unpaired Student’s *t* test, two-tailed *P* values < 0.05 being considered statistically significant. Variance was similar between groups.

Further details are provided in Supplementary Materials and Methods.

## Results

**Fig. 1:**
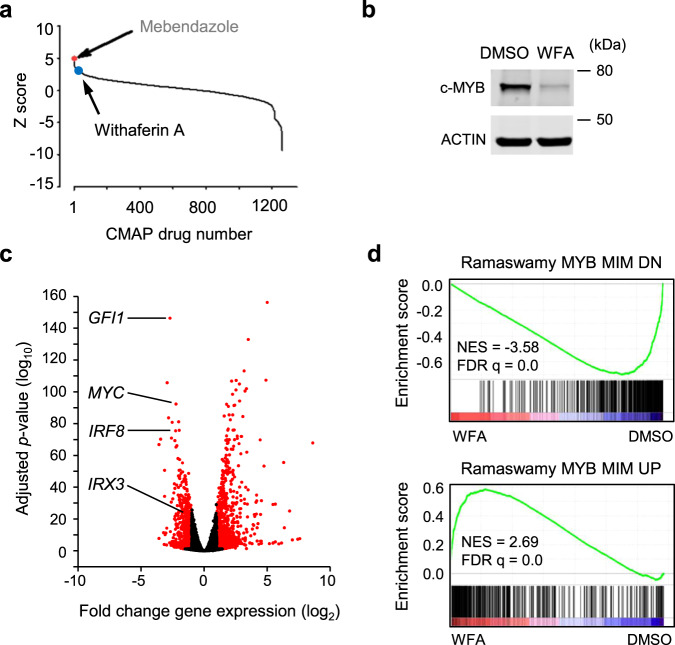
Identification of Withaferin A as a c-MYB directed therapeutic by Connectivity Map analysis. **a** 1309 Connectivity Map (CMAP) drugs ranked based on the significance of regression scores between their transcriptional profiles and that of the query from SPIEDw [33] interrogation of the CMAP database. The Z-score corresponds to the number of standard deviations of the score away from the mean. The position of Withaferin A (WFA, rank 15) is shown, with that of mebendazole [26] (rank 1) for reference. **b** Western blot example of c-MYB protein expression in THP1 AML cells after 6 h treatment with DMSO or 1 μM WFA. **c** Volcano plots of RNA-seq analysis showing fold gene expression changes in THP1 cells following treatment with DMSO or 1 μM WFA for 6 h. Expression changes greater than 2-fold and *P* < 0.05 are shown in red, Wald test. Positions of c-MYB target genes *GFI1*, *MYC*, *IRF8* and *IRX3* are indicated. **d** GSEA demonstrating enrichment of genes downregulated or upregulated, following peptidomimetic targeting of c-MYB function in AML cells [25], in the WFA induced gene expression changes.

We previously published the identification of mebendazole as a c-MYB targeting drug in AML cells [26] through interrogation of the CMAP database [32] with a c-MYB gene signature using the SPIEDw web tool [33]. Mebendazole activity was dependent on the induction of c-MYB protein loss. Further analysis of the top hits identified by this screen demonstrated that the steroidal lactone Withaferin A (WFA) also induced rapid loss of c-MYB protein in THP1 AML cells (Fig. 1a, b). RNA-seq was performed to determine whether this effect was reflected in global gene expression changes upon short-term exposure of THP1 cells to WFA. Similar to mebendazole, WFA treatment reduced the expression of known c-MYB target genes (Fig. 1c and Supplementary Fig. 1). GSEA confirmed that WFA reversed both the activation and repression of c-MYB target genes (Fig. 1d and Supplementary Fig. 2).

**Fig. 2:**
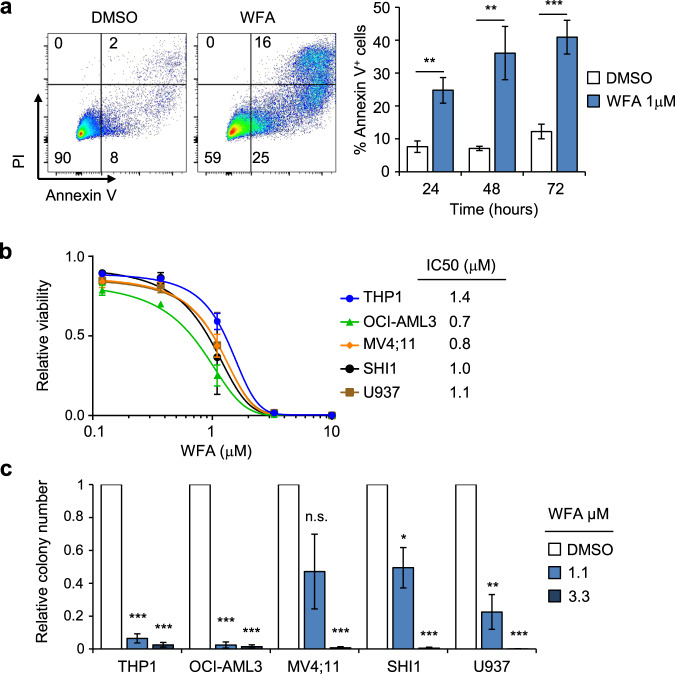
WFA exhibits robust anti-AML activity. **a** Apoptosis induction in THP1 cells following exposure to WFA. Example of Annexin V/PI staining in THP1 cells treated with DMSO or 1μM WFA for 72 hours (left panel) and quantification of Annexin V^+^ THP1 cells at indicated timepoints after exposure to DMSO or 1 µM WFA (right panel). Bars and error bars represent means and SD from three independent experiments. **P < 0.01; ***P < 0.001, unpaired Student’s *t*-test between DMSO and WFA treated cells. **b** Viability, normalized to DMSO controls, of AML cell lines treated for 72 h with indicated WFA concentrations. Indicated IC50 were calculated from non-linear regression for each cell line. Bars and error bars are means and SD of three independent experiments, each in triplicate. **c** Colony formation, normalized to DMSO controls, of indicated AML cell lines in the presence of indicated WFA concentrations. Bars and error bars are means and SD of three independent experiments. *P < 0.05; **P < 0.01; ***P < 0.001; n.s. not significant, one sample *t* test.

**Fig. 3:**
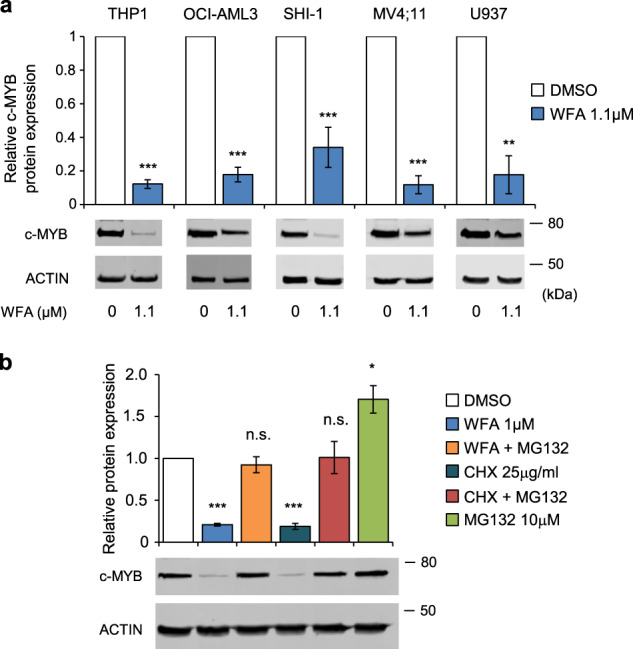
WFA induces c-MYB protein ablation. **a** c-MYB protein expression in AML cells after 6 hours treatment with DMSO or WFA, normalized to actin loading control and to DMSO treated controls. Bars and error bars are means and SD of three independent experiments. **P < 0.01; ***P < 0.001, one sample *t*-test. Western blots below graphs show examples of c-MYB protein expression. **b** c-MYB protein expression in THP1 cells treated for 6 h with indicated combinations of DMSO, WFA (1 µM), MG132 (10 µM), cyclohexamide (CHX) (25 µg/ml), normalized to actin loading control and to DMSO treated controls. Bars and error bars are means and SD of three independent experiments. *P < 0.05; ***P < 0.001; n.s. not significant, one sample *t*-test. Western blots below graphs show examples of c-MYB protein expression.

Exposure of THP1 cells to WFA resulted in cell death (Fig. 2a). WFA also exhibited potent anti-leukemia activity against a panel of AML cell lines, with IC50s in the range 0.7–1.4 μM (Fig. 2b) and inhibited their colony-forming activity (Fig. 2c). This was accompanied by reduced c-MYB protein levels in all cell lines tested (Fig. 3a). Next, we examined whether WFA had an impact on the expression of other transcription factors and co-factors known to be associated with c-MYB in AML cells [16]. Exposure of THP1 cells to WFA for 6 h resulted in moderately reduced SPI1 expression but had no significant effect on LYL1 and CBP expression levels (Supplementary Fig. 3). To determine whether c-MYB loss was an indirect result of loss of AML cell viability or reduced proliferation, we examined cell death and cell cycle following 6 h WFA exposure. No significant induction of apoptosis was observed at this early time-point after WFA exposure and addition of the pan-caspase inhibitor ZVAD had no effect on c-MYB loss, indicating that the latter did not result from cell death induction (Supplementary Fig. 4a). Furthermore, WFA-induced changes in cell cycle were only apparent after 24 h exposure and were not observed at 6 h (Supplementary Fig. 4b–e).

Proteasomal inhibition with MG132 was found to block WFA-induced c-MYB loss, indicating that protein degradation plays a major role in this process (Fig. 3b and Supplementary Fig. 5). However, c-MYB protein levels did not reach those in cells treated with MG132 alone and cycloheximide resulted in a similar pattern of c-MYB protein expression (Fig. 3b). This suggests that reduced production of c-MYB may also contribute to c-MYB loss. It is important to note that WFA also reduced *c-MYB* gene expression in THP1 cells (Supplementary Fig. 6a), as seen previously with mebendazole [26]. To examine whether reduced *c-MYB* transcription could explain the effect of WFA on c-MYB protein levels, THP1 cells were treated with WFA in the presence or absence of actinomycin D. Despite similar inhibition of *c-MYB* gene expression after 4 h exposure, c-MYB protein levels were significantly lower in cells treated with WFA and actinomycin D than actinomycin D alone (Supplementary Fig. 6). This is consistent with WFA-induced c-MYB loss being a result of independent effects on c-MYB protein degradation and *c-MYB* gene expression.

**Fig. 4:**
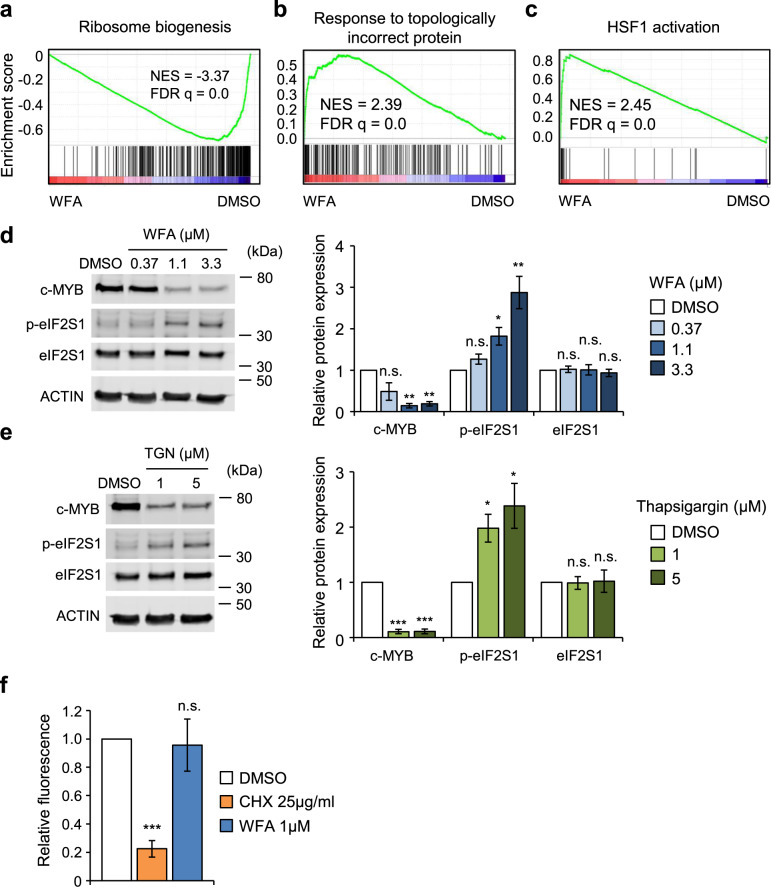
c-MYB ablation by WFA is associated with induction of the unfolded protein response in AML cells. **a**–**c** GSEA of gene sets associated with **a** ribosome biogenesis, **b** unfolded protein response and **c** HSF1 activation in gene expression changes in THP1 cells following treatment with DMSO or 1 μM WFA for 6 hours. **d**, **e** Example (left panel) and quantification (right panel) of c-MYB, phospho-eIF2S1 and total eIF2S1 expression in THP1 cells treated for 6 h with the indicated concentrations of **d** WFA and **e** thapsigargin. Data are normalized to actin loading control and to DMSO treated controls. Bars and error bars are means and SD of three independent experiments. *P < 0.05; **P < 0.01; ***P < 0.001; n.s. not significant, one sample *t*-test. **f** OPP incorporation in THP1 cells treated with 25 µg/ml CHX or 1 µM WFA for 4 h, normalized to DMSO treated control. Bars and error bars are means and SD of three independent experiments. ***P < 0.001; n.s. not significant, one sample *t*-test.

A recent study demonstrated that WFA induces phosphorylation of the translation initiating factor eIF2S1 and subsequent inhibition of protein translation in T acute lymphoblastic leukemia cells [34]. Indeed, GSEA of WFA induced gene expression changes demonstrated negative enrichment of genes associated with ribosome biogenesis (Fig. 4a). In contrast, positive enrichment was found for gene sets associated with induction of the unfolded protein response (UPR) and with activation of the proteotoxic stress inducible transcription factor HSF1 (Fig. 4b, c and Supplementary Fig. 7). Interestingly, heat shock was shown previously to cause a decrease in the rate of c-MYB protein synthesis [35]. Together, these analyses suggest that c-MYB loss may result from WFA-induced global translational inhibition. Indeed, we found that WFA caused a dose-dependent increase in eIF2S1 phosphorylation in THP1 cells, similar to that induced by the endoplasmic stress inducer thapsigargin (Fig. 4d, e), suggesting that its effect on c-MYB protein levels may be mediated by translational inhibition. However, analysis of O-propargyl-puromycin (OPP) incorporation into newly synthesized protein in THP1 cells after exposure to WFA for 6 h did not detect any significant change in global protein translation, in contrast to the rapid inhibition following cycloheximide treatment (Fig. 4f). Inhibition in OPP incorporation was evident only after prolonged 24 h WFA exposure (Supplementary Fig. 8). These data suggest that inhibition of global protein translation cannot explain c-MYB loss at early time-points after WFA exposure. However, it is important to note that these experiments do not rule out selective effects of WFA on translation of *c-MYB* messenger RNA.

**Fig. 5:**
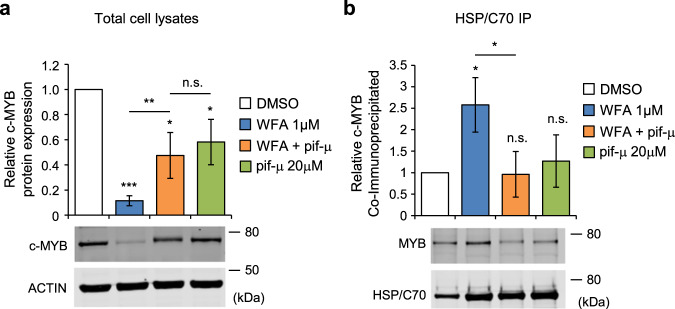
WFA exposure increases association between c-MYB and HSP70/HSC70. **a** c-MYB protein expression in THP1 cells treated for 6 hours with 1 µM WFA or 20 µM pifithrin-µ, alone or in combination, normalized to actin loading control and to DMSO treated controls. Bars and error bars are means and SD of four independent experiments. *P < 0.05; **P < 0.01; ***P < 0.001; n.s. not significant, one sample *t*-test, and unpaired Student’s *t t*est between single and combina*t*ion treatments. Western blots below graph show examples of c-MYB protein expression. **b** c-MYB protein co-immunoprecipitated with anti-HSP70/HSC70 from THP1 cells treated for 6 hours with 1 µM WFA or 20 µM pifithrin-µ, alone or in combination. Bars and error bars are means and SD of three independent experiments. *P < 0.05; n.s. not significant, one sample t test, and unpaired Student’s t test between single and combination treatments. Western blots below graph show examples of c-MYB (top) co-immunoprecipitated with HSP70/HSC70 (bottom).

We previously reported that mebendazole caused c-MYB loss in AML cells by disrupting the HSP70/HSC70 chaperone complex [26]. Pharmacological targeting of the HSP70/HSC70 nucleotide-binding domain, or heat shock protein synthesis, also caused loss of c-MYB, demonstrating the importance of this chaperone complex for maintenance of c-MYB protein levels. Since WFA was found to induce gene expression changes associated with the UPR and HSF1 activation, we decided to investigate the possible involvement of the HSP70/HSC70 chaperone complex in c-MYB loss. Inhibition of HSP70/HSC70 by pifithrin-μ was found to partially rescue loss of c-MYB induced by WFA (Fig. 5a). Pifithrin-μ (also known as 2-phenylethynesulfonamide or PES) targets HSP70 and HSC70 by covalent modification of cysteine residues in the substrate-binding domain [36–38]. However, pifithrin-μ thiol reactivity may also result in inhibition of other proteins in addition to the HSP70/HSC70 complex. To further investigate this, we examined the effect of WFA and pifithrin-μ on association of c-MYB with HSP70/HSC70. In contrast to our previous analyses with mebendazole, WFA treatment caused an increase in c-MYB bound to the HSP70/HSC70 chaperone complex (Fig. 5b). However, increased binding was reversed by addition of pifithrin-μ (Fig. 5b). Thus, c-MYB association with HSP70/HSC70 inversely correlated with total c-MYB protein expression levels. These data are consistent with the possibility of c-MYB degradation being mediated via the HSP70/HSC70 chaperone complex in response to WFA exposure and the resulting proteotoxicity.

**Fig. 6:**
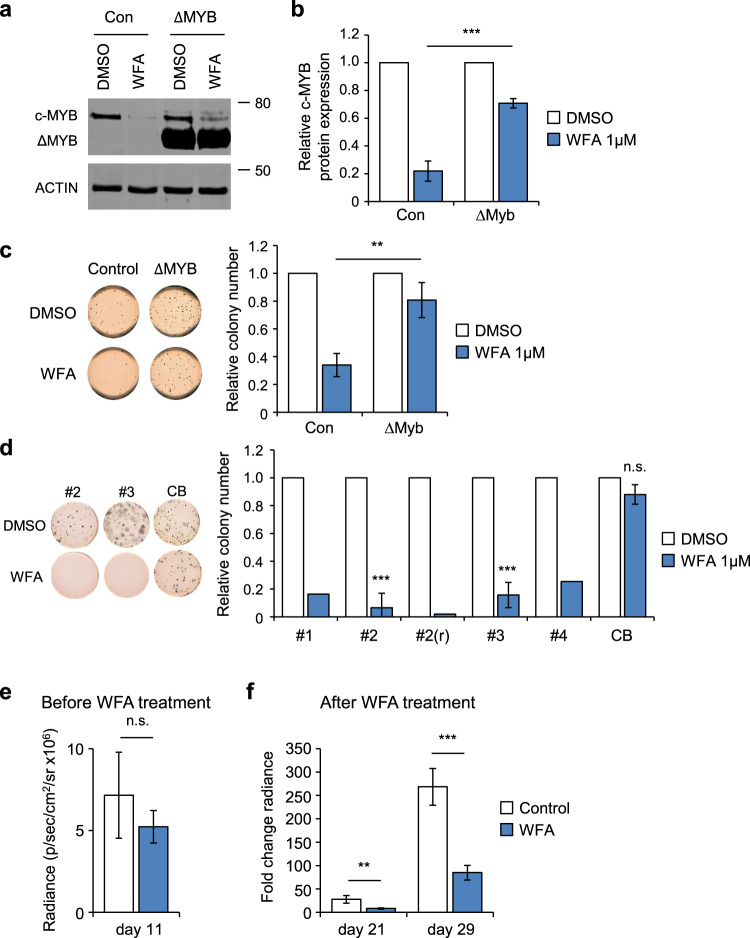
WFA exhibits c-MYB dependent anti-AML activity, inhibits AML PDX colony formation and impairs AML progression in vivo. **a**, **b** Example **a** and quantification **b** of c-MYB protein expression in empty vector (Con) or c-MYB deletion mutant (ΔMYB) transduced THP1 cells, 8 h after treatment with DMSO or 1 µM WFA, normalized to actin loading control and to DMSO treated controls. Bars and error bars are means and SD of three independent experiments. ***P < 0.001, unpaired Student’s *t* test between WFA treated Con and ΔMYB THP1 cells. **c** Example (left panel) and quantification, normalized to DMSO controls, (right panel) of colony formation by Con or ΔMYB transduced THP1 cells after pre-treatment with DMSO or 1 μM WFA. Cells were treated with DMSO or WFA for 8 hours, washed, and placed into methylcellulose culture. Bars and error bars are means and SD of three independent experiments. **P < 0.01, unpaired Student’s *t*-test between WFA trea*t*ed Con and ΔMYB THP1 cells. **d** Example (left panel) and quantification, normalized to DMSO controls, (right panel) of colony formation by AML PDX samples [#1, #2, #2(r), #3 and #4] and normal CD34^+^ cord blood (CB). Bars are means of one [#2(r)] and two (#1 and #4) independent experiments, and bars and error bars are means and SD of three (#2, #3, and CB) independent experiments. ***P < 0.001; n.s. not significant, one sample *t*-test. **e**, **f** Bioluminescence signal (radiance = photons/s/cm^2^/steradian) in NSG recipient mice **e** 11 days after THP1-LUC2 cell inj**e**ction and before WFA treatment, and **f** fold change in bioluminescence signal 21 and 29 days after injection, following onset of treatment with control or WFA-containing diet at day 11. **P < 0.01; ***P < 0.001; n.s. not significant, unpaired Student’s *t*-test between control and WFA treated groups. Bars and error bars are means and SD of values form control (*n* = 4) and WFA treated (*n* = 4) groups.

Next, we examined the importance of c-MYB modulation by WFA with respect to its anti-AML activity. GSEA of WFA-induced gene expression changes in THP1 cells revealed negative enrichment of the leukemia stem cell (LSC) self-renewal signature (Supplementary Fig. 9a), consistent with the reported importance of c-MYB in maintaining the AML LSC self-renewal program [13] and our previous data with mebendazole [26]. Indeed, short-term exposure of THP1 cells to WFA significantly impaired colony formation (Fig. 6a–c). To determine whether this was due to c-MYB loss, a stabilized c-MYB mutant (ΔMYB) [39] was expressed from a heterologous promoter in THP1 cells, thus making it less susceptible to ablation by WFA. Expression of ΔMYB was found to partially protect THP1 colony formation following transient WFA exposure (Fig. 6a–c), similar to the rescue we observed previously in THP1 cells following mebendazole treatment [26]. These data are consistent with a previous report that used a reporter screen to identify WFA as an inhibitor of c-MYB transcriptional activity, which induced differentiation of HL60 AML cells [40]. However, in contrast to this report, we did not detect gene expression changes in THP1 cells exposed to WFA consistent with C/EBPβ inhibition, rather being more consistent with activation of this transcription factor (Supplementary Fig. 9b). These data indicate that WFA inhibits AML colony formation by inducing loss of c-MYB protein, consequently impairing AML self-renewal.

We then examined whether the anti-AML activity of WFA could be extended to AML patient-derived xenograft (PDX) cells. Exposure of AML PDX samples to WFA resulted in significant inhibition of colony formation (Fig. 6d). Interestingly, WFA sensitivity was found to be unaltered in a relapsed AML PDX sample, when compared to the original PDX. In contrast, WFA had no significant effect on colony formation by normal CD34^+^ cord blood cells (Fig. 6d and Supplementary Fig. 10), suggesting the existence of a therapeutic window for the anti-AML activity of WFA. In order to determine whether this activity has potential for clinical translation, we next examined the effect of oral WFA administration on AML disease progression in vivo. Indeed, longitudinal bioluminescence imaging demonstrated that WFA treatment significantly impaired disease progression in NSG mice engrafted with THP1 AML cells (Fig. 6e, f).

## Discussion

The essential function of c-MYB in AML [41, 42] makes it an attractive target for development of novel therapeutics and drug repositioning [16, 22, 24, 25]. We previously reported identification of mebendazole as a c-MYB-directed anti-AML therapeutic, via CMAP screening [26]. Here we have characterized the anti-AML activity of another top hit from this screen, the steroidal lactone WFA. Our data demonstrate that WFA exposure rapidly induces c-MYB ablation in AML cells, leading to loss of cell viability, reduced colony formation and impaired disease progression in vivo. AML colony formation could be rescued partially by over-expression of a stabilized ΔMYB mutant, supporting a causal association of c-MYB ablation with the anti-AML activity of WFA. The anti-AML activity of WFA extended to AML PDX samples, while no toxicity was evident for normal CD34^+^ cord blood cells. These data position WFA, alongside mebendazole, as a promising candidate for repositioning into AML therapy.

Our data demonstrate that WFA acts on c-MYB at the protein level and on *c-MYB* gene expression. In this context it is notable that inhibition of c-MYB function in AML cells, via peptidomimetic disruption of c-MYB:CBP/P300 complex formation, was reported to result in decreased *c-MYB* gene expression [25]. Furthermore, we also previously demonstrated reduced *c-MYB* gene expression upon mebendazole-induced c-MYB protein ablation [26]. The impact of both WFA and mebendazole on *c-MYB* gene expression were evident before any effects on cell cycle were detectable. These data are consistent with previous reports that c-MYB can positively regulate its own gene expression in hematopoietic cells [43].

The mechanism underlying WFA induced c-MYB protein ablation appears to depend on its induction of proteotoxicity and the UPR and consequent disruption of c-MYB homeostasis by the HSP70/HSC70 chaperone complex. Induction of proteotoxicity, the UPR and heat shock response pathways in AML cells by WFA is consistent with data from a previous study that used an HSF1-reporter screen to identify anti-cancer compounds capable of inducing the endogenous heat shock response [44]. Many of the diverse natural compounds identified by this study, including WFA and the pentacyclic triterpenoid Celastrol, contained the α,β-unsaturated carbonyl motif, suggesting that anti-cancer activity of these compounds could in part depend on induction of proteotoxicity and disruption of protein homeostasis by the thiol-reactive moieties. Although the targets of these compounds mediating proteotoxicity remain to defined, in this mechanism, thiol-reactivity would represent the generic effector motif with target selectivity conferred by the diverse structural backbones [44].

We reported previously that c-MYB targeting by mebendazole was also associated with HSP70/HSC70 chaperone complex disruption [26]. This suggests that the relatively short half-life of c-MYB protein in AML cells makes it particularly vulnerable to disturbance of its chaperone-mediated turnover. However, in contrast to our previous studies with mebendazole, WFA-induced c-MYB ablation correlated with increased binding of c-MYB to HSP70/HSC70, suggesting that this association was involved in targeting c-MYB for degradation following WFA-induced proteotoxicity. Indeed, removal of protein aggregates from the cytosol and degradation of damaged and misfolded proteins is one of the central mechanisms through which the HSP70/HSC70 complex ensures the quality of cellular proteins [45–47]. Despite the undoubted pleiotropic effects of both WFA [48] and mebendazole, it is their impact on c-MYB protein levels that likely explains their effectiveness at eliminating AML cells, due to the essentiality of this transcription factor in AML.

However, it is possible that inhibition of MYB and consequent loss of AML cell viability may occur via multiple mechanisms. Abrogation of the MYB transcriptional program by WFA may also occur through inhibition of co-operating transcription factors, such as the reduced expression levels of SPI1 observed in this study. Furthermore, other thiol-reactive natural compounds, such as Celastrol [49] and the sesquiterpene lactones (STL) parthenolide [50], mexicanin-1 [50] and 4,15-iso-atriplicolide tiglate (AT) [51] have also been shown to inhibit the transcriptional activity of MYB. Thus, it is possible that inhibition of the MYB transcriptional program by WFA occurs simultaneously at several different levels.

Despite the relatively poor reported bioavailability of WFA [52], oral administration significantly impaired AML progression in engrafted mice. This is consistent with previous reports of in vivo WFA activity in numerous disease models [27, 53], including a wide range of cancer types [54]. Thus, although further development of clinical formulations may be necessary, the safety and efficacy profiles of WFA position it as an exciting novel anti-AML therapeutic.

## Supplementary information

Supplementary Figures.

Supplementary Methods.

Supplementary Figure Legends.

Supplementary Table 1.
