# Supplementary material for: Identification of a c-MYB-directed therapeutic for acute myeloid leukemia
Source: Leukemia. 2022 Apr 2;36(6):1541–9. doi: 10.1038/s41375-022-01554-9 (PMC9162920; doi:10.1038/s41375-022-01554-9)
Supplement: Supplementary file 1 — Supplementary Figures. [file 41375_2022_1554_MOESM1_ESM.pdf]

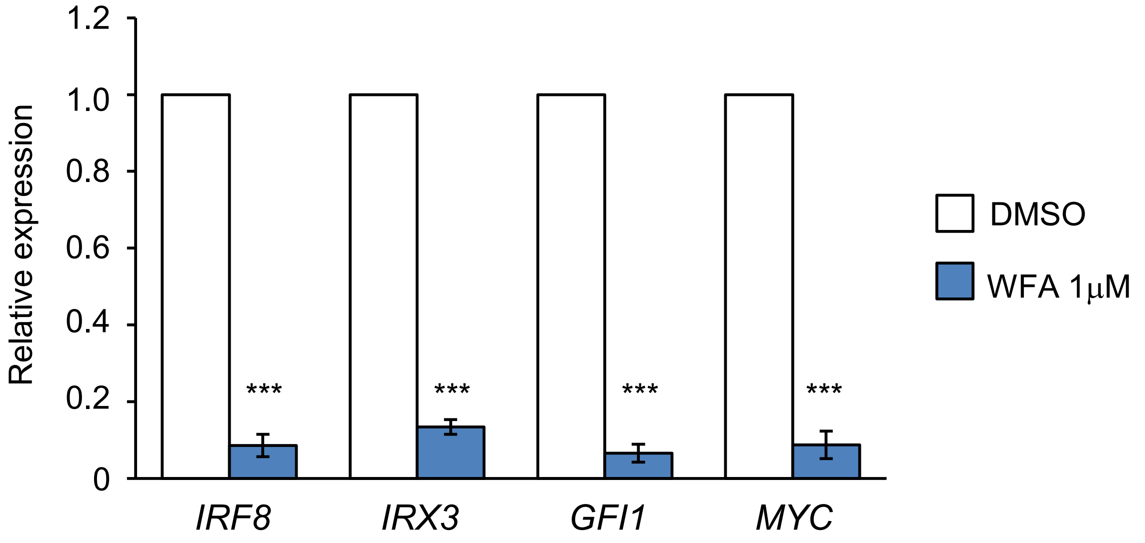

Supplementary Figure 1

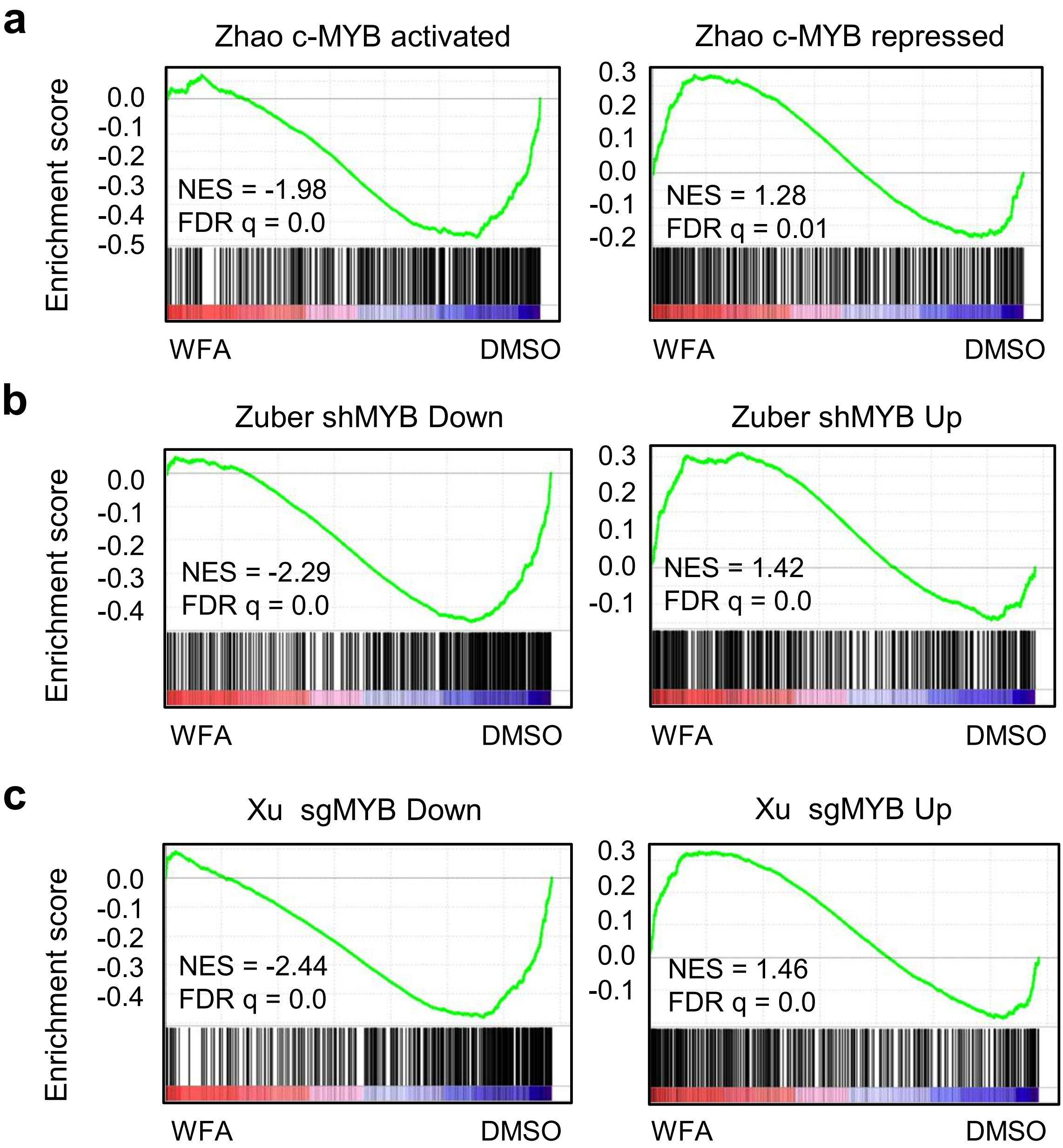

Supplementary Figure 2

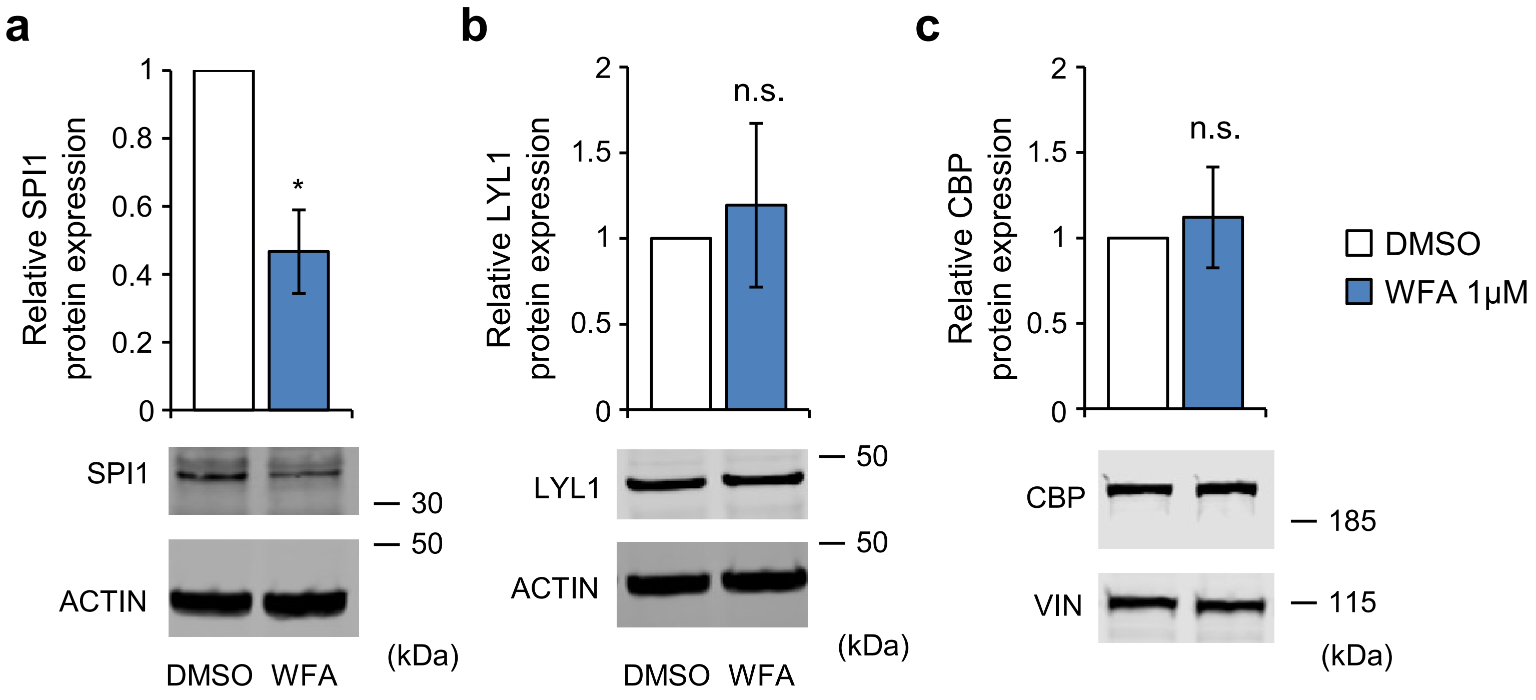

Supplementary Figure 3

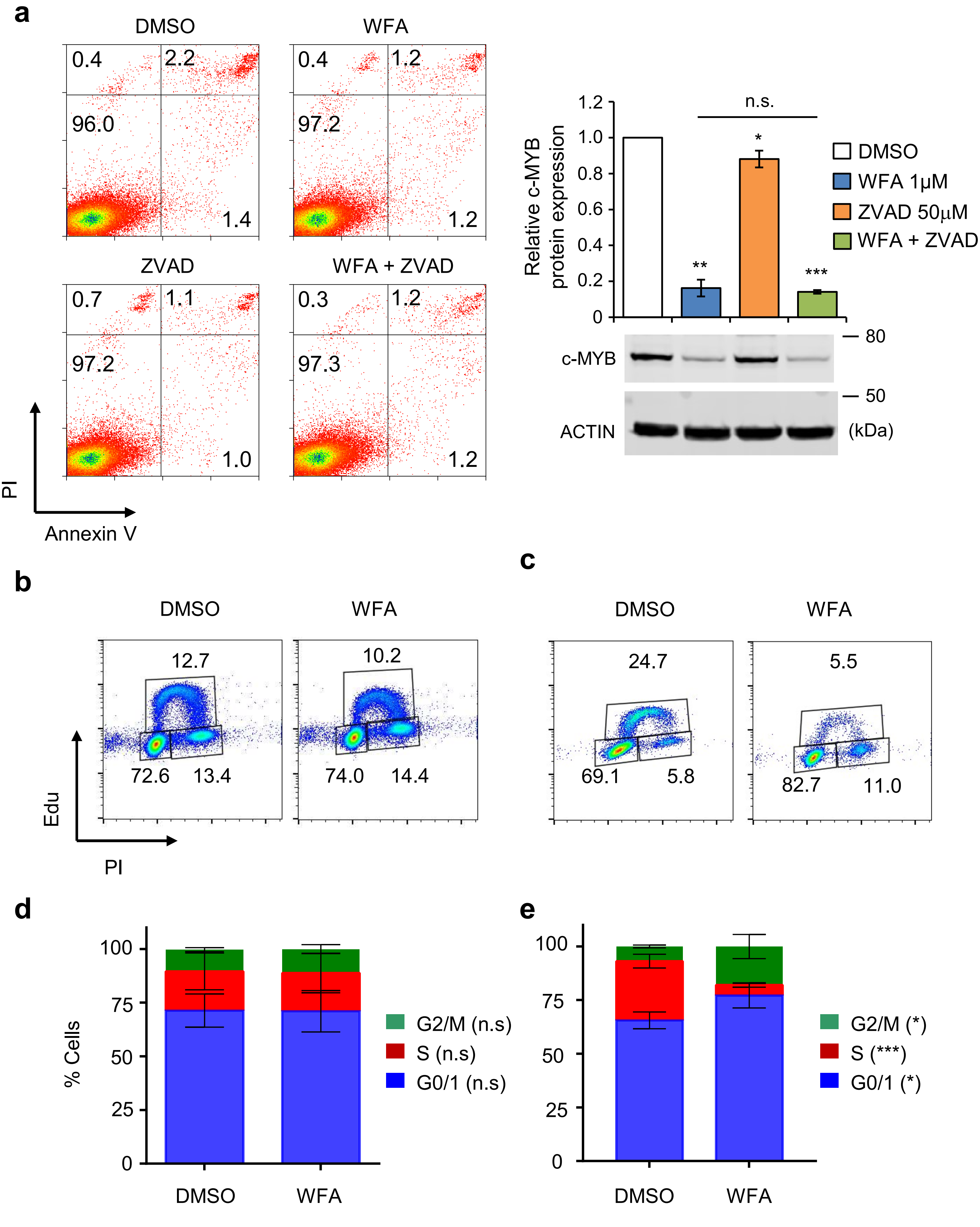

Supplementary Figure 4

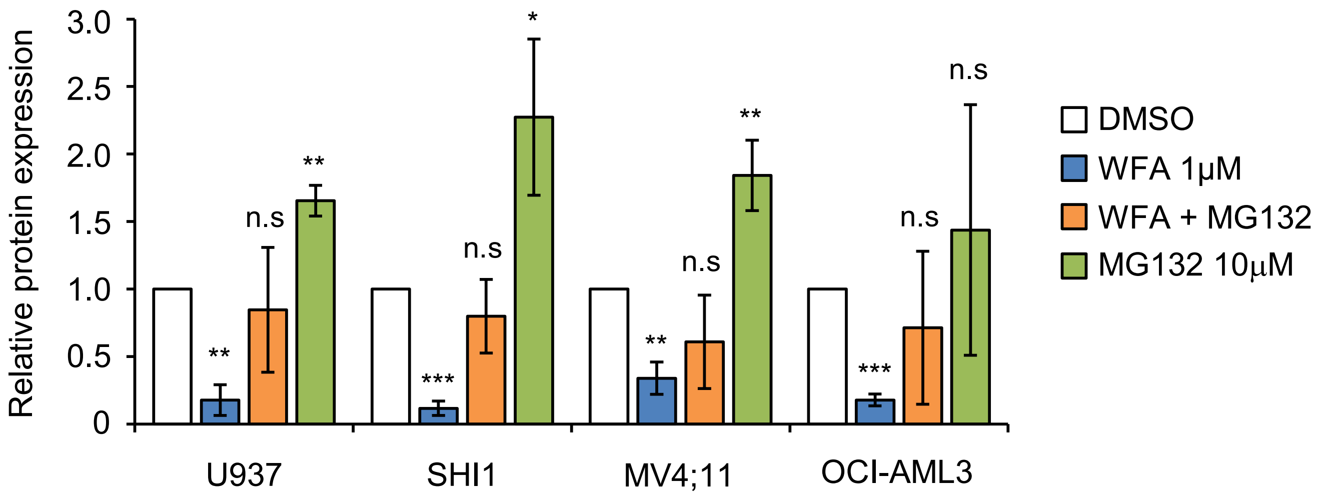

Supplementary Figure 5

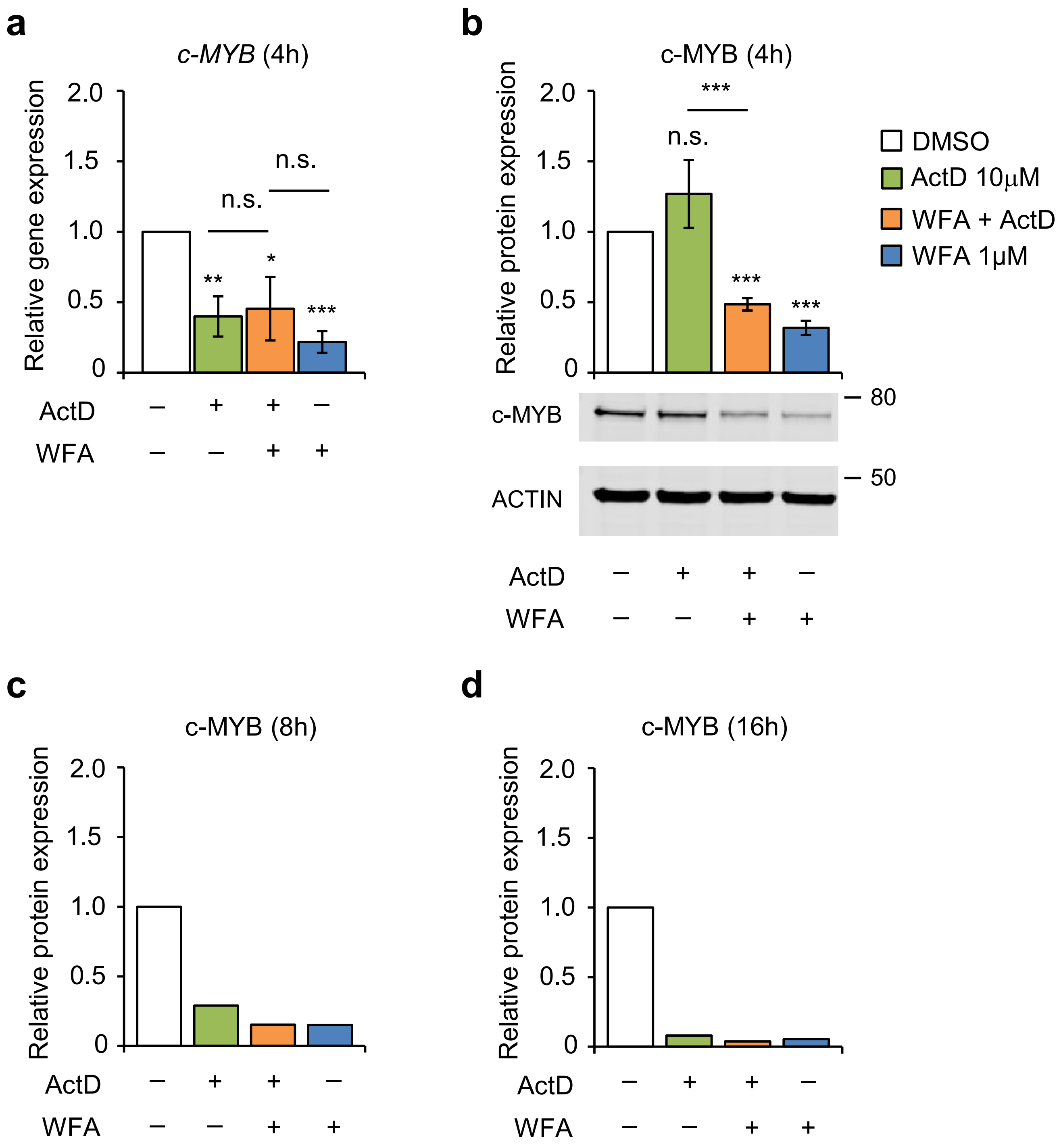

Supplementary Figure 6

Top five C5 GO BP (UP & DN) and C2 Reactome (UP) gene sets enriched in WFA RNA-seq data

c5.go.bp.v7.4.symbols_DN		NES	FDR q
1	GOBP_RIBOSOME_BIOGENESIS	-3.37	0
2	GOBP_RRNA_METABOLIC_PROCESS	-3.3	0
3	GOBP_NCRNA_PROCESSING	-3.13	0
4	GOBP_NCRNA_METABOLIC_PROCESS	-3.13	0
5	GOBP_RIBOSOMAL_LARGE_SUBUNIT_BIOGENESIS	-3.07	0
**c5.go.bp.v7.4.symbols_UP**		**NES**	**FDR q**
1	GOBP_RESPONSE_TO_TOPOLOGICALLY_INCORRECT_PROTEIN	2.39	0
2	GOBP_CHAPERONE_MEDIATED_PROTEIN_FOLDING	2.38	0
3	GOBP_DE_NOVO_PROTEIN_FOLDING	2.22	0
4	GOBP_CHAPERONE_COFACTOR_DEPENDENT_PROTEIN_REFOLDING	2.2	0
5	GOBP_PROTEIN_REFOLDING	2.2	0
**c2.cp.reactome.v7.4.symbols_UP**		**NES**	**FDR q**
1	REACTOME_ATTENUATION_PHASE	2.46	0
2	REACTOME_HSF1_ACTIVATION	2.45	0
3	REACTOME_HSF1_DEPENDENT_TRANSACTIVATION	2.4	0
4	REACTOME_HSP90_CHAPERONE_CYCLE_FOR_STEROID_HORMONE…	2.38	0
5	REACTOME_CELLULAR_RESPONSE_TO_HEAT_STRESS	2.1	0.001

Supplementary Figure 7

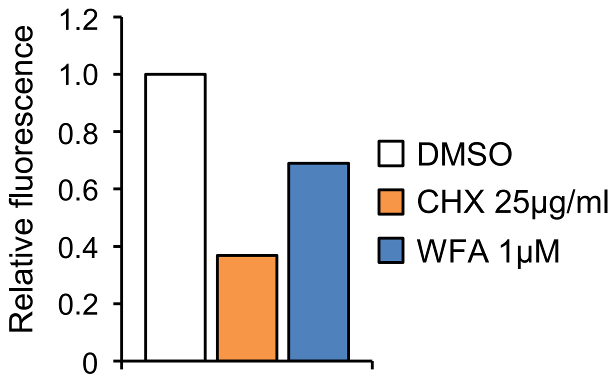

Supplementary Figure 8

a

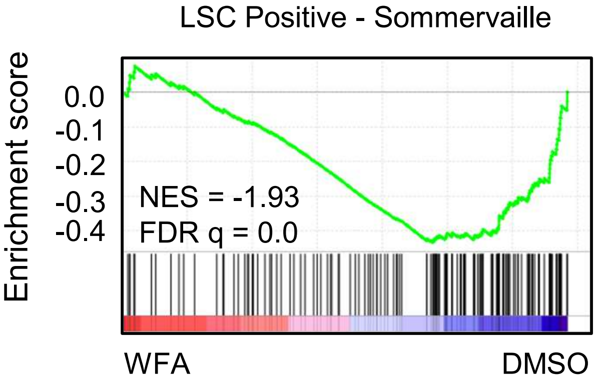

b

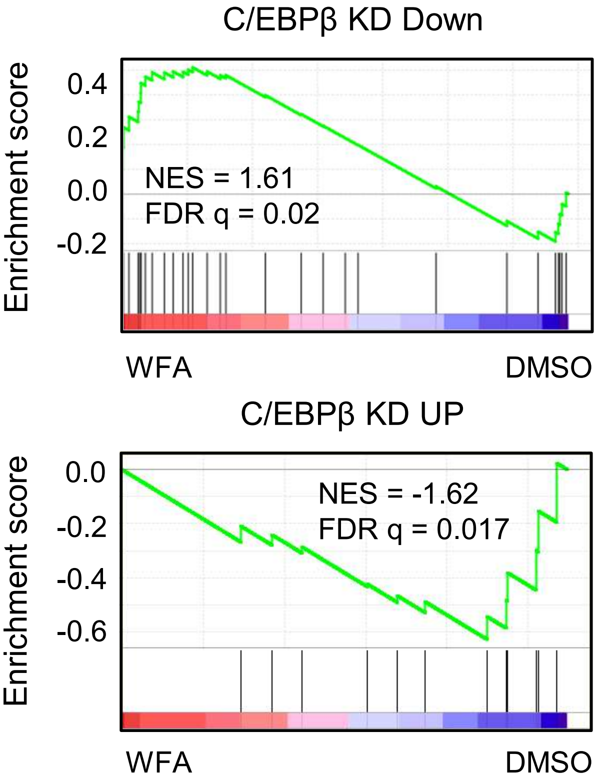

Supplementary Figure 9

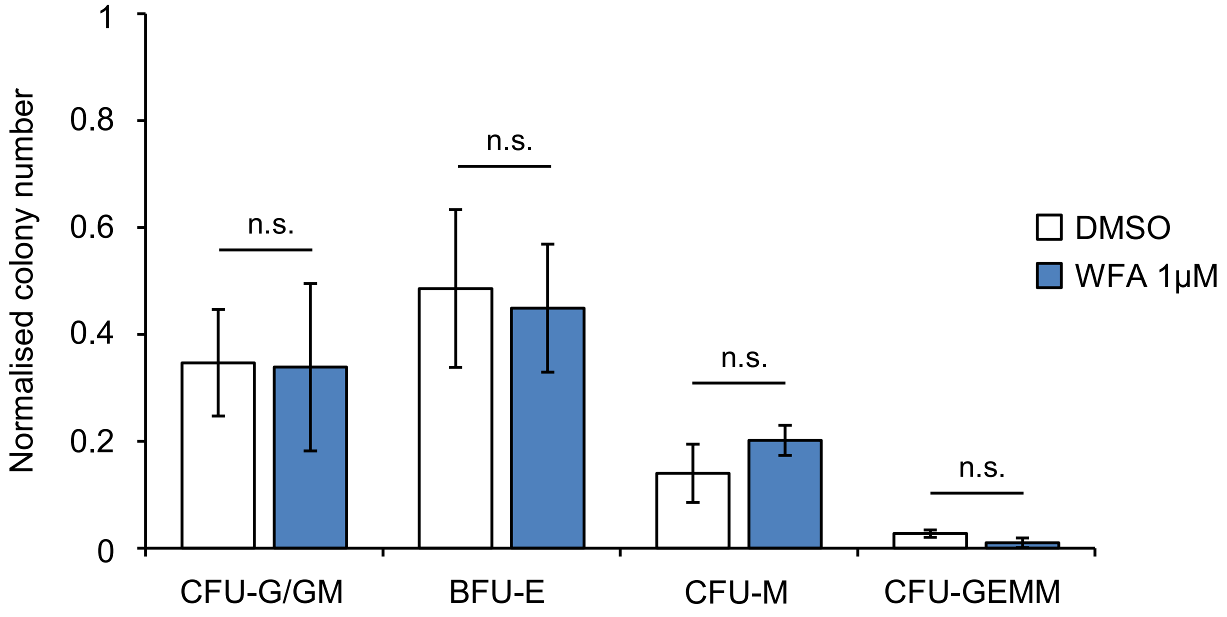

Supplementary Figure 10
