# Supplementary material for: Identification of a c-MYB-directed therapeutic for acute myeloid leukemia
Source: Leukemia. 2022 Apr 2;36(6):1541–9. doi: 10.1038/s41375-022-01554-9 (PMC9162920; doi:10.1038/s41375-022-01554-9)
Supplement: Supplementary file 2 — Supplementary Methods. [file 41375_2022_1554_MOESM2_ESM.pdf]

Supplementary Materials and Methods

Cell culture

Human AML cell lines were cultured in RPMI or IMDM (SHI-1) supplemented with 10–20% FCS, L-glutamine and penicillin/streptomycin. 293FT (ThermoFisher Scientific, Hemel Hempstead, UK) cells were maintained in DMEM with 10% FCS, L-glutamine, penicillin/streptomycin and 500 µg/ml G418.

In vivo transplantation

6-10 week old female NOD-SCID-$\gamma^{-/-}$ (NSG; The Jackson Laboratory, Bar Harbor, ME, USA) mice were used for transplantation. Group sizes were chosen based on previous estimates of disease latency in transplanted mice and experiments in the literature performing similar studies. No samples or animals were excluded from analysis.

RNA sequencing (RNA-seq)

Total cellular RNA was extracted using the RNeasy Plus Mini Kit (Qiagen) according to manufacturer's instructions. 100 ng of RNA per sample were analysed using Bioanalyser 2100 (Agilent Technologies, Santa Clara, CA) to verify RNA integrity prior to amplification. Samples were processed using the Illumina TruSeq RNA sample prep kit Version2 (p/n RS-122-2001) according to manufacturer's instructions (Illumina, Cambridge, UK). Briefly, mRNA was selected using paramagnetic dT beads and fragmented by metal hydrolysis to approximately 150 bp lengths. Random primed cDNA was then generated and adapters compatible with Illumina sequencing were ligated before being enriched by 12 cycles of PCR. Libraries were quantified, normalised and pooled before sequencing on an Illumina NextSeq 500, generating approximately 20 million 43 bp read pairs per sample. Fastq was then

demultiplexed and generated using Illumina bcl2fastq v2.19 before pre-processing (trimmomatic) to remove adapter read-through and poor quality sequences. Pre-processed data were then aligned to the genome (UCSC hg38) with Bowtie 2 and deduplicated using Picard. Reads-per-transcript were counted by FeatureCounts, before differential expression analysis by SARTools, a DESeq2 wrapper. All tools were invoked through the Galaxy Project for NGS analysis.

Western blot and co-immunoprecipitation (Co-IP) analysis

The following primary antibodies were used for western blot analysis: anti-c-MYB (clone 1-1, Merck Millipore, Watford, UK) and (EPR718(2), Abcam, Cambridge, UK), anti-β-Actin (C4, Santa Cruz Biotechnology, Texas, USA), anti-EIF2S1 (ab13762, Abcam) and anti-phospho-EIF2S1 (ab131505, Abcam), anti-SPI1 (NBP2-27163, Novus Biologicals, Colorado, USA), anti-LYL1 (GTX129329, GeneTex International Corporation, California, USA), anti-CBP (PA1-847, ThermoFisher Scientific), and anti-HSP70/HSC70 (H-300, Santa Cruz Biotechnology). Cells were lysed in reducing sample buffer (100 mM dithiothreitol, 2% sodium dodecyl sulfate, 10 % glycerol, 0.002 % bromophenol blue, 62.5 mM Tris-HCL pH 6.8). For Co-IP analyses, anti-HSP70/HSC70 (W27, Santa Cruz Biotechnology) was used. Cells were washed twice with cold PBS, lysed and proteins immunoprecipitated using the Pierce Classic Magnetic IP/Co-IP Kit (ThermoFisher Scientific). Protein samples were resolved on gels 8 or 10 % polyacrylamide (0.36 M bis-Tris, 8-10 % acrylamide/bis) in MOPS-SDS running buffer (50 mM Tris, 50 mM MOPS, 1 mM EDTA, 0.1 % SDS). Gels were transferred onto nitrocellulose (LI-COR Biosciences, Cambridge, UK) membranes. Proteins were detected using appropriate IRDye 800CW and IRDye 680RD labelled secondary antibodies (LI-COR Biosciences). Quantification was performed on fluorescent images using the Odyssey® CLx and Image Studio software (LI-COR Biosciences).

Growth, apoptosis and cell cycle assays

The effect of WFA on growth of AML cell lines was examined by exposing them to different concentrations of WFA in liquid culture, starting at 1.25-2.5 x 10^5 cells/ml, and 72 hours later were stained with TO-PRO®-3 (ThermoFisher Scientific) stain and total viable cells were determined by flow cytometry analysis on a BD FACSArray™ Bioanalyzer, using Summit 4.3 software (Beckman Coulter, High Wycombe, UK). Apoptosis was detected using the Annexin V Apoptosis detection kit (eBioscience, Hatfield, UK). Cell cycle analysis was performed using the Click-iT EdU Alexa Flour 647 Flow Cytometry Assay Kit (Invitrogen, Life Technologies). Cells were analyzed on an LSRII (BD Bioscience, San Jose, CA, USA), and the data were analyzed with Summit 4.3 software (Beckman Coulter).

Protein synthesis

Total protein synthesis with the Protein Synthesis Assay kit (Cayman Chemical) according to the manufacturer's instructions. OPP incorporation was analysed by flow cytometry.
