# Supplementary material for: Identification of a c-MYB-directed therapeutic for acute myeloid leukemia
Source: Leukemia. 2022 Apr 2;36(6):1541–9. doi: 10.1038/s41375-022-01554-9 (PMC9162920; doi:10.1038/s41375-022-01554-9)
Supplement: Supplementary file 3 — Supplementary Figure Legends. [file 41375_2022_1554_MOESM3_ESM.pdf]

Supplementary Figure Legends

Supplementary Figure 1. Relative expression of c-MYB target genes in THP1 cells exposed to DMSO or 1μM WFA for 6 hours. The RNA was collected from experiments independent to those used for the RNA-seq analysis. Bars and error bars are means and SD of three independent experiments. ***$P < 0.001$, one sample *t* test.

Supplementary Figure 2. GSEA of **a** c-MYB repressed and activated gene sets, bound by c-MYB in mouse myeloid ERMYB cells [28] and deregulated in THP1 cells following siRNA mediated c-MYB silencing [29]; and **b** shRNA [15] and **c** CRISPR-mediated [30] c-MYB targeting in AML cells, in RNA-seq analysis of THP1 cells treated for 6 hours with DMSO or 1μM WFA.

Supplementary Figure 3. a SPI1, **b** LYL1 and **c** CBP protein expression in THP1 cells after 6 hours treatment with DMSO or 1μM WFA, normalized to **a**, **b** actin or **c** vinculin (VIN) loading controls and to DMSO treated controls. Bars and error bars are means and SD of three independent experiments. *$P < 0.05$; n.s. not significant, one sample *t* test. Western blots below graphs show examples of protein expression.

Supplementary Figure 4. a Example of Annexin V/PI staining (left panel) and quantification of c-MYB protein expression (right panel) in THP1 cells treated with DMSO, 1μM WFA, 50μM ZVAD or WFA + ZVAD for 6 hours. Bars and error bars are means and SD of three independent experiments. *$P < 0.05$; **$P < 0.01$; ***$P < 0.001$; n.s. not significant, one sample *t* test, and unpaired Student's *t* test between single and combination treatments. Western blots below graphs show examples of c-MYB protein expression. **b**, **c** Examples of cell cycle flow cytometry plots of THP1 cells treated for **b** 6 hours or **c** 24 hours with DMSO or 1μM WFA. Numbers inside plots are percentages of cells in G0/G1 (bottom left), S (top) and G2/M (bottom right) phases of the cell cycle. **d**, **e** Quantification of data from **b** and **c**. Bars and error bars are

means and SD of three independent experiments. *$P < 0.05$; ***$P < 0.001$; n.s. not significant, unpaired Student's *t* test between DMSO and WFA treated cells.

Supplementary Figure 5. c-MYB protein expression in AML cell lines treated for 6 hours with indicated combinations of DMSO, WFA (1µM) and MG132 (10µM), normalized to actin loading control and to DMSO treated controls. Bars and error bars are means and SD of three (U937) and four (SHI1, MV4;11, OCI-AML3) independent experiments. *$P < 0.05$; **$P < 0.01$; ***$P < 0.001$; n.s. not significant, one sample *t* test. Western blots below graphs show examples of c-MYB protein expression.

Supplementary Figure 6. a *MYB* gene expression and **b** c-MYB protein expression in THP1 cells 4 hours after treatment with indicated combinations of DMSO, WFA (1µM) and Actinomycin D (10µM). Eukaryotic 18S rRNA was used to quantify qRT-PCR data and c-MYB protein was normalized to actin loading control and to DMSO treated controls. Bars and error bars are means and SD of four independent experiments. *$P < 0.05$; **$P < 0.01$; ***$P < 0.001$; n.s. not significant one sample *t* test, and unpaired Student's *t* test between single and combination treatments. Western blots below graph show examples of c-MYB protein expression. **c**, **d** c-MYB protein expression in THP1 cells **c** 8 hours and **d** 16 hours after treatment with indicated combinations of DMSO, WFA and Actinomycin D. Data represent a single experiment.

Supplementary Figure 7. The table shows the enrichment of the top five down- and upregulated gene sets from the c5.go.bp.v7.4.symbols.gmt and the top five upregulated gene sets form the c2.cp.reactome.v7.4.symbols.gmt collection of gene sets from the MSigDB database (http://www.gsea-msigdb.org/gsea/msigdb/index.jsp), in gene expression changes resulting from 6 hours exposure of THP1 cells to 1 µM WFA or DMSO.

Supplementary Figure 8. OPP incorporation in THP1 cells treated with 25µg/ml CHX or 1µM WFA for 24 hours, normalized to DMSO treated control.

Supplementary Figure 9. GSEA of **a** gene sets positively correlating with leukaemia stem cell frequency and **b** shRNA targeting of C/EBPβ [29, 31], in RNA-seq analysis of THP1 cells treated for 6 hours with DMSO or 1µM WFA.

Supplementary Figure 10. Morphological assessment of colony formation by normal $CD34^+$ cord blood cells in the presence of DMSO or 1µM WFA. Colonies were scored as CFU-G/GM, BFU-E, CFU-M and CFU-GEMM. Bars and error bars are means and SD of three independent experiments. n.s. not significant, unpaired Student's *t* test between DMSO and WFA cultures.
