# Supplementary material for: Identification of a c-MYB-directed therapeutic for acute myeloid leukemia
Source: Leukemia. 2022 Apr 2;36(6):1541–9. doi: 10.1038/s41375-022-01554-9 (PMC9162920; doi:10.1038/s41375-022-01554-9)
Supplement: Supplementary file 4 — Supplementary Table 1. [file 41375_2022_1554_MOESM4_ESM.pdf]

Supplementary Table 1. Patient sample characteristics.

PDX	Patient id	Sex	Age (yrs)	Cytogenetics
#1	210915c	M	1.85	Derived chromosome 17 resulting in gain of 1q, del(6p), add(18p) and loss of chromosomes 1, 7, 8, 9, 11 and 15 in one of the doubled-up clones by G-banded analysis. FISH:TP53 was preserved
#2	280717b	F	2.38	Abnormal clone with a t(11;19)(q23;p13) translocation detected by G-banded analysis. Evidence of KMT2A rearrangement by interphase FISH analysis
#2(r)	270418a	F	3.08	Abnormal clone with a t(11;19)(q23;p13) translocation, as seen at diagnosis by G-banding. Somatic GATA2 mutation (new on relapse), FLT3-ITD negative.
#4	160119a		0.44	Monosomy 7 detected by interphase FISH and G-band analysis

MLL-AF9 #3: AML1547 patient sample characteristics previously reported in Walf-Vorderwulbecke et al (2018)
